# Adaptive Radiation within Marine Anisakid Nematodes: A Zoogeographical Modeling of Cosmopolitan, Zoonotic Parasites

**DOI:** 10.1371/journal.pone.0028642

**Published:** 2011-12-13

**Authors:** Thomas Kuhn, Jaime García-Màrquez, Sven Klimpel

**Affiliations:** 1 Biodiversity and Climate Research Centre (BiK-F, LOEWE), Medical Biodiversity and Parasitology; Senckenberg Gesellschaft für Naturforschung (SGN); Goethe-University (GO), Institute for Ecology, Evolution and Diversity, Frankfurt am Main, Germany; 2 Departamento de Gestión Ambiental, Carbones del Cerrejón Limited, Bogotá, Colombia; Biodiversity Insitute of Ontario - University of Guelph, Canada

## Abstract

Parasites of the nematode genus *Anisakis* are associated with aquatic organisms. They can be found in a variety of marine hosts including whales, crustaceans, fish and cephalopods and are known to be the cause of the zoonotic disease anisakiasis, a painful inflammation of the gastro-intestinal tract caused by the accidental consumptions of infectious larvae raw or semi-raw fishery products. Since the demand on fish as dietary protein source and the export rates of seafood products in general is rapidly increasing worldwide, the knowledge about the distribution of potential foodborne human pathogens in seafood is of major significance for human health. Studies have provided evidence that a few *Anisakis* species can cause clinical symptoms in humans. The aim of our study was to interpolate the species range for every described *Anisakis* species on the basis of the existing occurrence data. We used sequence data of 373 *Anisakis* larvae from 30 different hosts worldwide and previously published molecular data (n = 584) from 53 field-specific publications to model the species range of *Anisakis* spp., using a interpolation method that combines aspects of the alpha hull interpolation algorithm as well as the conditional interpolation approach. The results of our approach strongly indicate the existence of species-specific distribution patterns of *Anisakis* spp. within different climate zones and oceans that are in principle congruent with those of their respective final hosts. Our results support preceding studies that propose anisakid nematodes as useful biological indicators for their final host distribution and abundance as they closely follow the trophic relationships among their successive hosts. The modeling might although be helpful for predicting the likelihood of infection in order to reduce the risk of anisakiasis cases in a given area.

## Introduction

Approximately 20,000 cases of human anisakidosis infections are reported every year from a wide range of coastal regions, primarily in Japan and Europe [1]. This zoonosis, named after a family of marine nematodes, the Anisakidae (“whale-, seal-, cod-, herringworms”), is the result of the ingestion of infectious third stage larvae (L3) in raw or undercooked marine fish products. The term anisakidosis designates infections caused by nematodes of the family Anisakidae, whereas the term anisakiasis includes all infections that are caused by members of the genus *Anisakis* [2]. Besides the major clinical symptoms such as abdominal pain, nausea, vomiting and fever, anisakidosis infections can be associated with an increased risk of allergic responses caused by even very small doses of nematode antigens [2]. Increased globalisation and fast growing international markets, the massive demand for exotic dishes such as sushi, sashimi, salted/smoked herring, and the increasing propensity not to overcook food, have led to an accumulation of infections even in countries that do not have traditions of consuming raw fish [2], [3]. Besides the seal worms *Pseudoterranova decipiens* and *Contracaecum* spp. (diseases: anisakidosis), species of the genus *Anisakis* (*A. simplex* complex, *A. physeteris*) are considered the most common cause of human anisakiasis infections [1]. The general life cycle of these parasites involves a broad range of invertebrates, (such as crustaceans and chaetognaths), teleost fish species and cephalopods, which act as intermediate or paratenic hosts. Marine mammals such as cetaceans (toothed and baleen whales) and, sometimes, pinnipeds (seals) serve as final hosts [4], [5]. Toothed and baleen whales of the families Delphinidae, Ziphiidae, Physeteridae and Kogiidae are considered the main definitive hosts, but members of the Balaenopteridae, Pontoporidae, Monodontidae, Phocoenidae and Neobalaenidae have also been parasitised [6]. The increased number of individuals resulting from stronger regulation to protect whale populations is thought to be another reason for the increase in anisakiasis infections in the last three decades [1], [2].

In spite of the fact that *A. simplex* s.l. is the most common anisakiasis cause, it is still unclear whether all *Anisakis* species can cause clinical symptoms in humans [1]. This is why the identification of species and knowledge about their distribution is of primary importance for understanding parasite epidemiology, especially for species of medical, veterinary and commercial importance [7].

The distribution of marine helminth parasites is influenced by a wide range of abiotic factors such as hydrographic and climate conditions. It is also influenced by transmission pathways that closely follow the trophic relationship between the parasites' final, intermediate and transport hosts. Thus the species range and, especially, feeding behaviour of the hosts has to be taken into account in order to understand the occurrence of anisakid nematodes and predict the likelihood of infections in a given area [5].

Most descriptions of zoogeographical distribution patterns rely on sampling data from regions where the occurrence of species has been recorded [8]. However, this type of information is scarce for many species and usually biased towards accessible areas or places of special interest [9]–[11]. In order to tackle systematic errors such as oversimplification (e.g. outline maps) or underestimation (e.g. dot maps) in the assessment of biogeographical maps, several techniques were developed that interpolate and project distribution ranges to places where species potentially exist but have not yet been sampled. Triangulation techniques, for example, have played an important role in conservation, and the results of applying the minimum convex polygon to a set of species collection records have been a crucial point in assessing the conservation status of species [12], [13]. However, this method has gone through a series of refinements that seek to avoid certain constraints and biases (e.g. overestimation, detection of disjunctive distribution). Burgmann and Fox [14] recommend the use of the α-hull- instead of the convex-hull-interpolation technique because it produces a more flexible outer polygon surface that is more resistant to bias and allows the outer polygon line to be broken into detailed and discrete hulls. More recently, Raedig et al. [11] developed a geometric conditional triangulation approach that is based on a specified interpolation distance and avoids the problem of excluding narrow endemic species from the analysis. The final distribution range of a particular species is constructed not only on those areas that are included within the interpolated triangles, but also on those that connect species occurrence locations within the given interpolation distance as well as areas where locations are isolated due to a lack of neighbouring occurrences.

Based on these developments we present here a methodology for interpolating occurrence locations for every described *Anisakis* spp. (*Anisakis simplex* (Rudolphi, 1809, det. Krabbe, 1878) (sensu stricto), *A. pegreffii* Campana-Rouget & Biocca, 1955, *A. simplex* C (Nascetti et al., 1986, Mattiucci et al., 1997), *A. typica* (Diesing, 1860), *A. nascettii* Mattiucci et al., 2009, *A. ziphidarum* Paggi et al, 1998, *A. physeteris* Baylis 1923, *A. paggiae* Mattiucci et al. 2005, *A. brevispiculata* Dollfus, 1966), which combines aspects of the α-hull algorithm and the geometric interpolation approach [11], [15]–[25].

## Results and Discussion

A total of 373 *Anisakis* spp. larvae from 30 teleost and bony fish host species were collected during fieldwork in 21 different sampling areas and identified by sequence analyses of ribosomal internal transcribed spacers (ITS-1/2, 5.8S) (Table S1, Figure S1) (Genbank Accession number JN968593–JN968965). Sequence analyses identified five already described *Anisakis* species (*A. simplex* s.s., *A. pegreffii*, *A. simplex* C, *A. typica*, *A. physeteris*) (Table S2). We combined the sequence data obtained with occurrence reports (584 nematode individuals) taken from 53 revised publications [5], [6], [18]–[21], [23], [26]–[71] and, using a non-commercial geographical information system (QGIS) transferred them as presence data to a grid with a resolution of 1°×1° covering the globe. Based on the centroids of the quadrats, the α-hull was calculated for 25 different values ranging from 2 to 50 in a two-step interval using the alpha-hull package [72]. The final distribution of every species was visualized in a range map with a continuous colour gradient, in which the intensity of the colour increased with the probability of species occurrence in a certain area (Figure S2).

**Figure 1:**
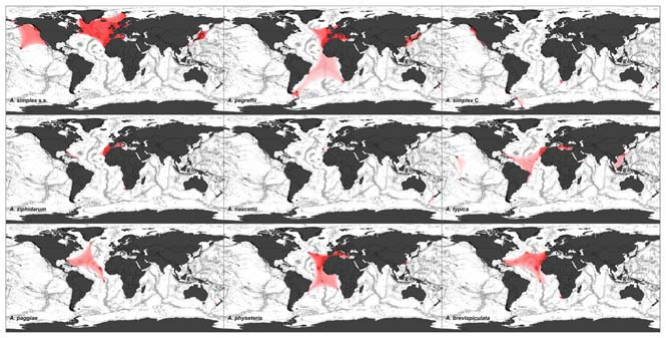
Modeled species range of every described *Anisakis* spp. Colour intensity reflects the probability of *Anisakis* occurrence. Dark red shadings indicate molecular proven *Anisakis* records. First clade: *Anisakis simplex* s.s., *A. pegreffii*, *A. simplex* C (building the *A. simplex* complex), *A. ziphidarum*, *A. nascettii* and *A. typica*. Second clade: *Anisakis paggiae*, *A. physeteris* and *A. brevispiculata* (*A. physeteris* complex) belong to the second clade (phylogeny follows Klimpel et al. 2010) [45].

The results of our modeling strongly indicate the existence of species-specific distribution patterns of *Anisakis* spp. within different climate zones and oceans that are in principle congruent with those of their respective final hosts (Figure 1). To date, 35 marine mammals and more than 75 different bony and elasmobranch fish species have been verified by molecular methods as potential hosts for *Anisakis* spp., but several studies provide evidence that the members of the *Anisakis* species complexes differ in their ecology and final host preferences [5], [21], [28], [37], [41], [73]. We found that species that are considered to be phylogenetically closely related show similar distribution patterns due to major congruities in the parasites' ecology and host preferences.

The genus *Anisakis* comprises two major clades. Whereas the first clade includes the *A. simplex* complex (*A. simplex* s.s., *A. pegreffii*, *A. simplex* C), as well as *A. typica* and both sister-species (*A. nascettii* and *A. ziphidarum*), the second clade consists exclusively of the *A. physeteris* complex (*A. brevispiculata*, *A paggiae*, *A. physeteris*) [5], [6].

Species of the *A. simplex* complex are mainly distributed within the Atlantic Ocean and in the West and East Pacific where large populations of their final hosts (Delphinidae) occur (Figure 1). In the case of *A. simplex* s.s., the most common exciter of human anisakiasis infection in Japan [74], the distribution could be narrowed down to a northern hemisphere range within the West and East Atlantic and Pacific Ocean, between 20°N and far beyond the Arctic polar circle (80°N) (Figure 1). The species range of *A. pegreffii* extends from Mediterranean waters through the East Atlantic Ocean down to the Antarctic Peninsula, with additional records in Japanese and Chinese waters (Figure 1). *Anisakis simplex* C has a discontinuous range: along the Canadian/US east coast; at the southern tip of Africa; and between New Zealand and Australia. Occurrence has also been proven between South American and Antarctic waters (Figure 1). These three closely related species of the *A. simplex* complex mainly include oceanic delphinids (e.g. Short-beaked Common Dophin *Delphinus delphis*, Long-finned Pilot Whale *Globicephala melas*) as final hosts in their life cycle, which are known to form large populations in the Atlantic Ocean where they usually feed on pelagic fish and cephalopods. The specific distribution patterns shown by these anisakids are likely to be a consequence of dispersal through the faeces of infested oceanic delphinids. This contrasts with the distribution of *A. typica*, the most abundant species in the tropics and subtropics between 45°N and 25°S (Figure 1). *Anisakis typica* is a common parasite of various dolphin species of warmer temperate waters, such as the Tucuxi *Sotalia fluviatilis*, the Common Bottlenose Dolphin *Tursiops truncatus* and the Pantropical Spotted Dolphin *Stenella attenuata*. These are more usually associated with shallower waters near the coast [50].

The sister taxa *A. ziphidarum* and *A. nascettii* are in principle congruent (Figure 1). While both species are distributed in the East Atlantic Ocean near Madeira and the Moroccan coast, the West Pacific between New Zealand and South Australia, and the southern tip of Africa, *A. ziphidarum* has a slightly extended range with additional records in the West Atlantic Ocean (Figure 1). This broadly similar zoogeography is also mirrored in their ecology as they have major similarities regarding their host preferences. Whereas *A. ziphidarum* also includes Cuvier's Beaked Whale *Ziphius cavirostris* as potential final host in its life cycle, both species seem to prefer ziphiid whales of the genus *Mesoplodon* spp. Taking into account the collection sites of the definitive hosts so far recorded, Mattiucci et al. [21] suggested that the geographical distribution of these parasites is related to that of their final hosts.

The close genetic relationship of the three representatives of the *A. physeteris* complex (*A. physeteris*, *A. paggiae*, *A. brevispiculata*) is also reflected in a relatively homogeneous distribution throughout the central Atlantic Ocean (Figure 1). Very similar to *A. ziphidarum* and *A. nascettii*, these species are known to be host specific and broadly include sperm whales as final hosts. While *A. physeteris* is the only species of this complex that parasitizes the Sperm Whale *Physeter macrocephalus*, all of them show clear preferences for the kogiid whales, the Pygmy Sperm Whale *Kogia breviceps* and the Dwarf Sperm Whale *Kogia sima*, which are mainly distributed in the central Atlantic Ocean. Sperm whales typically inhabit deepwater habitats in the tropics and the temperate zones where they mostly feed in depths between 500–1200 m, primarily on cephalopods and, less frequently, on deep sea fish and crustaceans [6], [75].

Our results support preceding studies that propose that anisakid nematodes as useful biological indicators for their final host distribution and abundance as they closely follow the trophic relationships among their successive hosts. Klimpel et al. (2011) [6] identified for the first time *A. paggiae* in the Irminger Sea and indicated a more extended migration towards northern latitudes than could have been inferred from the distribution range of kogiid whales reported so far. Our modeling clearly demonstrates, that the distribution patterns of *Anisakis* spp. can be narrowed down to certain areas within climatic zones and oceans and are mainly influenced by the species ranges and feeding behaviours of their respective intermediate and mammalian final hosts.

The world's least developed countries are particularly reliant on fish, which provides 27% of their dietary protein intake [76]. These countries also produce 20% of the world's fish exports [76]. A broad knowledge of the distribution of *Anisakis* spp. is of particular importance for predicting the likelihood of infection in order to reduce the risk of anisakidosis cases in a given area.

## Materials and Methods

### Ethics Statement

An approval by a review board institution or ethics committee was not necessary, because all the fish in the current study were obtained in different locations from fishermen selling fresh fish for consumption or were collected during regularly fishery cruises.

### Sample collection

A total of 373 *Anisakis* spp. larvae from 30 teleost and bony fish host species were collected during field phase from 21 different sampling areas (Table S1, Figure S1). Fish were identified according to Fishbase [77] and Gon and Heemstra [78]. Nematodes were extensively washed in 0.9% saline solution and identified morphologically to genus level according to Anderson (2000) and Moravec [79], [80]. All samples were fixed and stored in EtOH (abs.) prior to molecular examination.

### PCR amplification and species identification

Genomic DNA was isolated and purified from individual *Anisakis* spp. larvae using a genomic DNA extraction kit (Peqlab Biotechnology GmbH, Erlangen, Germany) according to the instructions of the manufacturer. The rDNA region comprising the ITS-1, 5.8S, ITS-2 and flanking sequences ( = ITS+) was amplified was amplified using primers NC2 (5′-TTA-GTT-TCT-TTT-CCT-CCG-CT-3′) and TK1 (5′-GGC-AAA-AGT-CGT-AAC-AAG-GT-3′) [81]. Primer TK1 was manually designed and then synthesized by Eurofins MWG Operon (Ebersberg, Germany). PCR-reaction (50 µl) included 25 µl Master-Mix (Peqlab Biotechnology GmbH, Erlangen, Germany) containing dNTP, MgCl_2_, Buffer and Taq-Polymerase, 3 µl of each primer, 14ddH_2_O and 5 µl genomic DNA. Each PCR reaction were performed in a thermocycler (Peqlab, Germany) under the following conditions: after an initial denaturation at 95°C for 1 min, 40 cycles of 94°C for 45 sec (denaturation), 55°C for 45 sec (annealing), 72°C for 45 sec (extension), followed by a final extension at 72°C for 10 min. Samples without DNA were included in each PCR run. PCR products were examined on 1% agarose gels. A 100 bp ladder marker (peqGOLD, Erlangen, Germany) was used to estimate the size of the PCR products. To prepare the samples for the sequencing, PCR products were purified with Cycle-Pure Kit (Peqlab Biotechnology GmbH, Erlangen, Germany). Afterwards a total volume of 7 µl, including 2 µl primer (individually) and 5 µl of the PCR product (250 ng/µl) were sequenced by Seqlab (Goettingen GmbH, Germany). Both spacers and the 5.8S gene from each PCR product were sequenced, using primer TK1 (5′-GGC-AAA-AGT-CGT-AAC-AAG-GT-3′). The ITS-1, 5.8S and ITS-2 sequences were determined for all 373 *Anisakis* nematodes isolated from the 30 host species (Genbank Accession number JN968593–JN968965). For species identification, the obtained sequences were compared with those previously deposited for the same marker in the Genbank using the BLASTn algorithm (Table S2).

### Zoogeographical interpolation

A database was built comprising the species names and the exact coordinates or geographical regions where they have been recorded. The spatial information were collected from the fieldwork and molecular analyses mentioned above as well as from literature review of 53 publications [5], [6], [18]–[21], [23], [26]–[71]. The geographical information contained in this database where transferred onto a grid of 1°×1° covering the globe in which species occurrences were overlaid and the corresponding quadrats marked as occurrences for each species in turn. This procedure was manually done using the non-commercial geographical information system Quantum GIS (QGIS, version 1.6.0) (Figure S2A) [82].

To estimate distribution ranges two steps were followed. First, the alpha-hull was calculated based on the centroids (i.e. points) of the quadrats where a species was recorded [14], [83]. For this, a Delauney triangulation between the centroids was applied and the average length of all lines of all triangles was calculated. The final hull (i.e. polygon) results from those centroids, which are connected by a line smaller than a multiple (alpha parameter) of the average line length (Figure S2B). This procedure was implemented using the ‘alphahull’ package [72] in the R statistical software [84]. Second, distribution ranges were defined as the corresponding quadrats intersecting the created polygons and also those quadrats with single occurrences that were not included within any of the polygons (Figure S2C) [11].

Since the size and number of polygons as well as the number of single occurrences depends on the value of the alpha parameter (i.e. an alpha of zero is equal to the set of single occurrences (dot map) and infinite alpha will encompass all points in one polygon (convex hull) [14], the distribution range of each species was calculated with 25 different alphas (i.e. a sequence of alphas ranging from 2 to 50 in a two-step interval). Results were summed up into a final distribution range map which is depicted in a continuous colour gradient. Darker colours represent areas where the probability of finding the species is relatively high and as the colours get lighter the uncertainties of an area being part of the species range increases (Figure S2D).

## Supporting Information

Figure S1 **Sampling locations of anisakid nematodes for molecular species identification.** Locations marked with red dots and respective locality abbreviations. Asterisks indicate multiple sampling sites. Abbreviations are listed in Table S1.
(TIF)

Figure S2 **Zoogeographical interpolation approach in four steps.** (A) geographical information where transferred onto a grid of 1°×1° covering the globe. Corresponding quadrats marked as occurrence for each species. (B) The final hull resulting from those centroids, which are connected by a line smaller than a multiple of the average line length (α-Parameter). (C) Distribution ranges were defined as the corresponding quadrats intersecting the created polygons. (D) Range was calculated for 25 different α-values ranging from 2 to 50 in a two-step interval. Results were visualized by a continuous colour gradient (Figure 1).
(TIF)

Table S1 **Information on parasites sampling locations and hosts.** Sampling locations of the 373 anisakid nematodes used for molecular analyses including abbreviation (Abb.), host and sample size (n). Asterisks mark multiple sampling sites with the same abbreviation (see Figure S1).
(XLS)

Table S2 **Total numbers of identified anisakid nematodes including hosts and sampling location.** For location abbreviations see Table S1. Asimss = *Anisakis simplex* s.s., Apeg = *A. pegreffii*, Atyp = *A. typica*, Aphy = *A. physeteris*.
(XLS)
