# Supplementary figures and images for: Adaptive Radiation within Marine Anisakid Nematodes: A Zoogeographical Modeling of Cosmopolitan, Zoonotic Parasites
Source: PLoS One. 2011 Dec 13;6(12):e28642. doi: 10.1371/journal.pone.0028642 (PMC3236750; doi:10.1371/journal.pone.0028642)

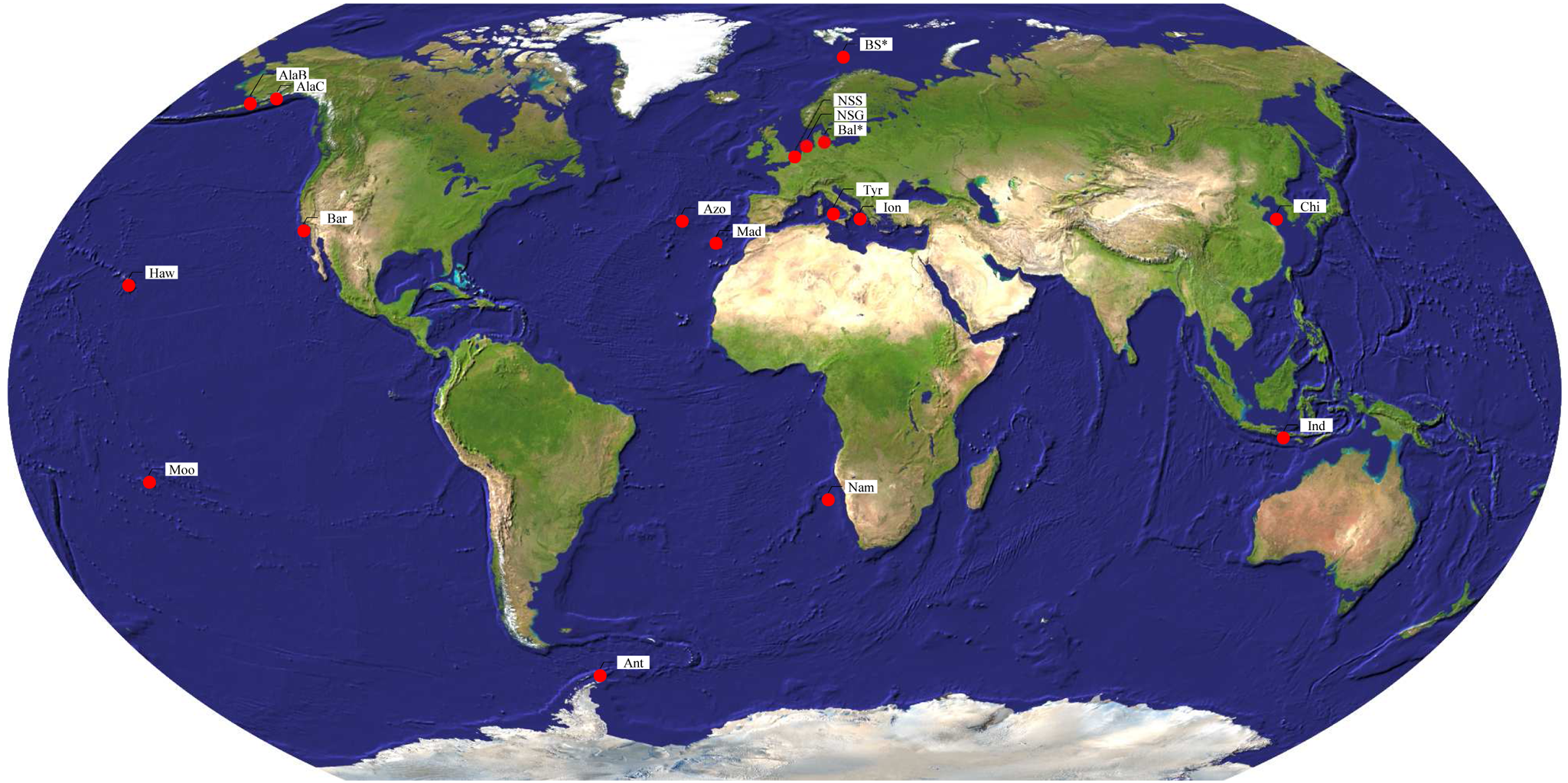

Supplement: Figure S1 — Sampling locations of anisakid nematodes for molecular species identification. Locations marked with red dots and respective locality abbreviations. Asterisks indicate multiple sampling sites. Abbreviations are listed in Table S1. (TIF) [file pone.0028642.s001.tif]

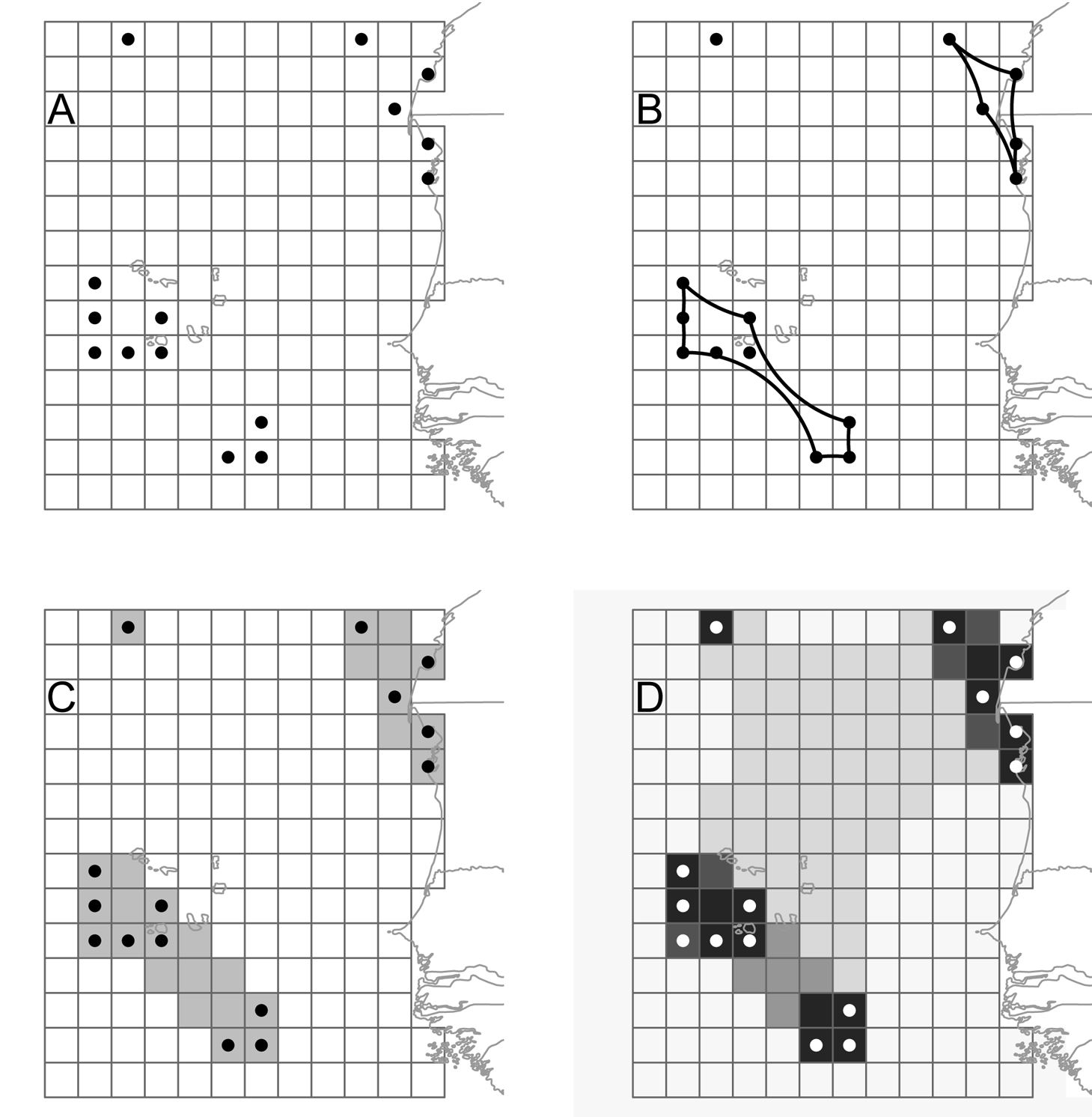

Supplement: Figure S2 — Zoogeographical interpolation approach in four steps. (A) geographical information where transferred onto a grid of 1°×1° covering the globe. Corresponding quadrats marked as occurrence for each species. (B) The final hull resulting from those centroids, which are connected by a line smaller than a multiple of the average line length (α-Parameter). (C) Distribution ranges were defined as the corresponding quadrats intersecting the created polygons. (D) Range was calculated for 25 different α-values ranging from 2 to 50 in a two-step interval. Results were visualized by a continuous colour gradient (Figure 1). (TIF) [file pone.0028642.s002.tif]
